# On Campus HIV Self-Testing Distribution at Tertiary Level Colleges in Zimbabwe Increases Access to HIV Testing for Youth

**DOI:** 10.1016/j.jadohealth.2022.09.004

**Published:** 2022-10-12

**Authors:** Grace McHugh, Andrea Koris, Victoria Simms, Tsitsi Bandason, Lovemore Sigwadhi, Getrude Ncube, Shungu Munyati, Katharina Kranzer, Rashida A. Ferrand

**Affiliations:** a Biomedical Research and Training Institute, Harare, Zimbabwe; b Duke Global Health Institute, Duke University, Durham, North Carolina; c MRC International Statistics and Epidemiology Group, London School of Hygiene & Tropical Medicine, London, UK; d AIDS and TB Unit, Ministry of Health and Child Care, Harare, Zimbabwe; e Department of Clinical Research, London School of Hygiene & Tropical Medicine, London, UK; f Division of Infectious Diseases and Tropical Medicine, University Hospital, LMU Munich, Munich, Germany

**Keywords:** HIV self-test, Youth, Tertiary educational institutions

## Abstract

**Purpose:**

HIV self-testing allows youth to access testing outside of healthcare facilities. We investigated the feasibility of peer distribution of HIV self-testing (HIVST) kits to youth aged 16–24 years and examined the factors associated with testing off-site rather than at distribution points.

**Methods:**

From July 2019 to March 2020, HIVST kits were distributed on 12 tertiary education campuses throughout Zimbabwe. Participants chose to test at the HIVST distribution point or off-site. Factors associated with choosing to test off-site and factors associated with reporting a self-test result for those who tested off-site were investigated using logistic regression.

**Results:**

In total, 5,351 participants received an HIVST kit, over 129 days, of whom 3,319 (62%) tested off-site. The median age of recipients was 21 years (interquartile range 20–23); 64% were female. Overall, 2,933 (55%) returned results, 23 (1%) of which were reactive. Being female (adjusted odds ratio [aOR] 1.16, 95% confidence interval [CI] 1.03–1.31), living on campus (aOR 1.24, 95% C11.09–1.40), used a condom at last sex (aOR 1.44, 95% C11.26–1.65), and previous knowledge of HIVST (aOR 1.22, 95% CI 1.09–1.37) were associated with off-site testing. Attending a vocational college and teachers training college compared to a university was associated with choosing to return results for those who tested off-site (OR 2.40, 95% CI 1.65–3.48, *p* < .001).

**Discussion:**

HIVST distribution is an effective method of reaching a large number of youth over a short period of time. Efforts to increase awareness and roll out of HIVST on campuses should be coupled with support for linkage to HIV prevention and treatment services.

Although rates of HIV infection have generally declined globally, youth remain at disproportionately high risk of HIV infection [1]. In sub-Saharan Africa, adolescent girls and young women aged 15–24 years make up 10% of the population, but accounted for one in four of all new HIV infections in 2019 [1]. Youth are less likely to undergo testing and have knowledge of their HIV status; data from a national population-based survey in Zimbabwe conducted in 2015–2016 showed that 59% of young people aged 15–24 years had ever had an HIV test compared to 86% of adults aged 35–39 years [2].

In high HIV prevalence settings, provider-initiated HIV testing and counselling has been recommended for many years [3]. Judgmental attitudes from providers and fear of stigma and discrimination often deter youth from accessing HIV testing in health facilities [4,5]. In recent years, HIV self-testing (HIVST) kits has become more widely available in sub-Saharan Africa, following evidence that individuals could test themselves correctly using oral mucosal transudate (OMT) tests, and that this was an acceptable approach with minimal risk of social harm [6]. HIVST offers autonomy and an opportunity to test oneself in a “safe space,” and appeals to groups, such as men and youth, that have been traditionally difficult to reach through facility-based HIV testing approaches. A growing evidence base demonstrates that youth welcome the opportunity to learn their HIV status if testing access is convenient and privacy and confidentiality can be provided, as with self-testing [6–11].

HIVST may help to increase HIV testing rates among youth, if provided in convenient locations. This may foster a habit of repeat testing of those at ongoing risk of infection. HIVST is a method of HIV testing delivery recommended by World Health Organization in high HIV prevalence settings [12].

A key service delivery question is how best to provide HIVST to hard-to-reach groups. We investigated the feasibility of provision of HIVST by peer distributors to 16–24 year olds on campuses of tertiary educational institutions across Zimbabwe. Specifically, we investigated the uptake of on-campus versus offsite HIVST and factors associated with off-site testing and subsequent linkage to HIV care.

## Methods

### Study setting

**Figure 1:**
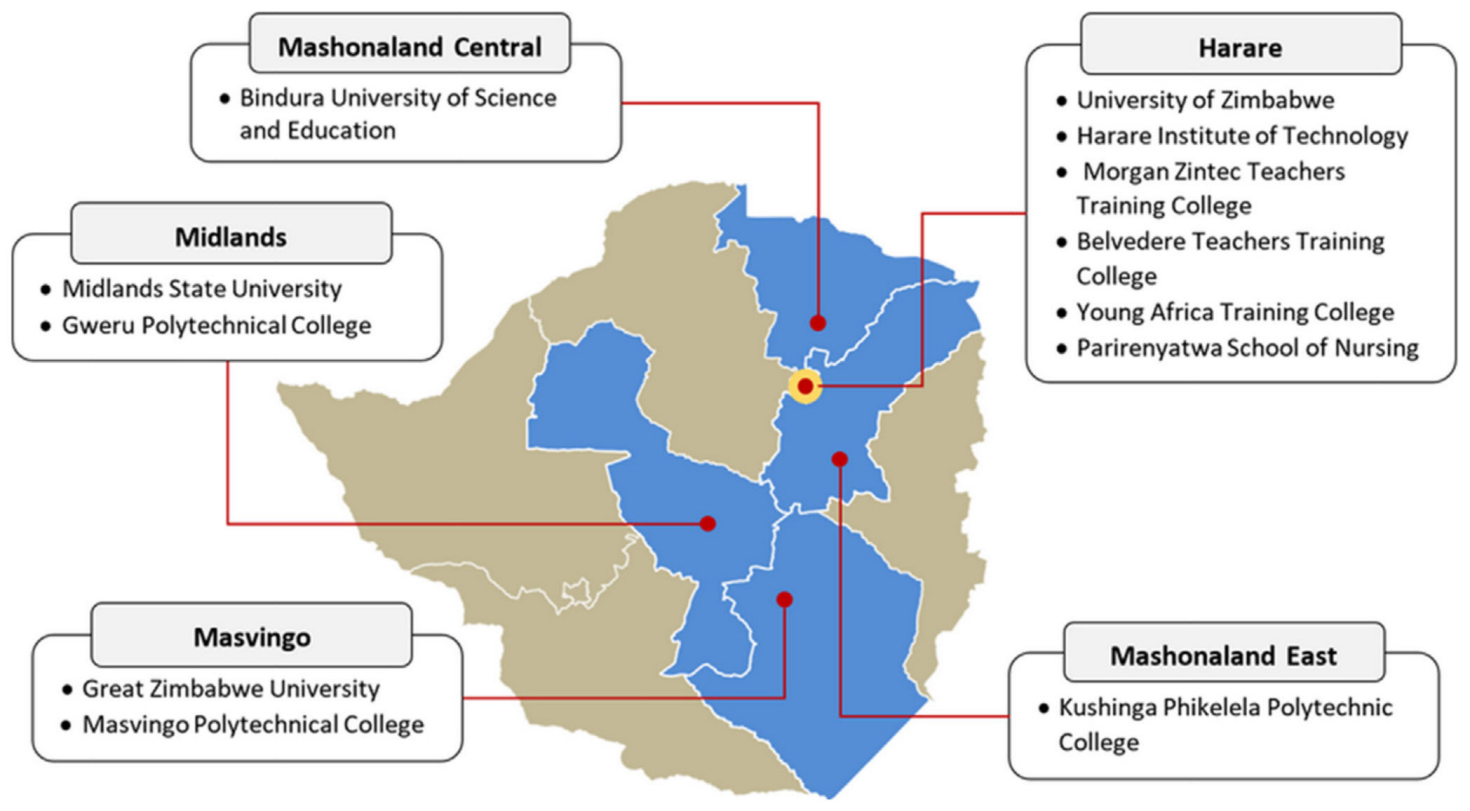
Study site locations in Zimbabwe.

A cross-sectional study was performed at 12 tertiary level educational institutions in five towns and cities, across four provinces in Zimbabwe between July 2019 and March 2020 (Figure 1). Institutions included universities (n = 4), polytechnical and technical training colleges (n = 5), teacher training colleges (n = 2), and one school of nursing. The minimum entry requirement in Zimbabwe to attend third level education (e.g., a polytechnical college) is a pass rate in at least five subjects in state examinations, taken after 4 years at secondary level education. Entry to university requires at least two pass grades in state examinations after 6 years of secondary education. Tuition fees for technical colleges start at approximately 50 USD per semester, university tuition fees are 300 USD per semester, and tuition at School of Nursing is free. All but one college had clinics located on or close to campus, 6 (50%) of whom offered facility-based HIV testing; of these, four also offered HIV treatment. None of the campus clinics offered HIVST. All institutions had a policy of inviting nongovernmental organizations to offer campusbased HIV testing campaigns, during the annual college calendar. Some of these campaigns included HIVST in the past.

### Study procedures

Institutions outside of Harare were chosen if they had a large student population and were geographically easily accessible for the research team. Universities and colleges within Harare were chosen based on the numbers of students possible to reach through the HIVST campaign. A team of eight peer distributors chose a distribution location at each campus following a social mapping exercise in collaboration with staff and students. The distributors walked around a campus for 2–3 days mapping landmarks which were then superimposed onto Field Maps, a mapping software program. Peer distributors shared the maps with students and staff, discussed the daily flow of student movement across campus and common gathering spaces. Following these discussions, the optimal distribution point was agreed upon. Distribution of kits, at a static tented site on campus, occurred between 8 A.M. and 4.30 P.M. on weekdays over term-time. Information booklets were provided on voluntary medical male circumcision (VMMC) and condoms. Information on clinics nearby that offered free sexually transmitted infection testing, family planning services and VMMC was also provided. Deans of institutions, student body representatives, and nursing staff at campus clinics were sensitized regarding the planned HIVST distribution.

### HIV testing

In Zimbabwe, HIV testing is available free of charge, traditionally provided by health facilities. Community-based testing is not a distribution model commonly used. The age of consent for HIV testing in Zimbabwe is 16 years of age. HIVST is included in the national HIV testing guidelines, and is usually offered within a clinical setting, i.e., the client does not take the test kit away. If the test is reactive, a confirmatory blood-based rapid diagnostic test is offered. HIVST is also available at private pharmacies throughout Zimbabwe and costs approximately 15 USD.

In this study, OMT (OraQuick, Orasure Technologies, USA) HIVST kits were offered to youth aged between 16 and 24 years if they had not had an HIV test in the previous 3 months and were not known to be living with HIV. HIV pretest information was provided to consented participants in groups of up to 10. Participants were given verbal instruction on how to use the kit. A pictorial demonstration of HIVST contained within the package contained instructions in English as well as the vernacular languages Shona and Ndebele. Participants were given the OMT kit and any questions or concerns were addressed by the peer distributors. Participants had the option to test on-site unassisted in one of four private tents, or to take the kit away with them to test at a time and location of their choice. This on-site testing option was offered to accommodate those who were anxious about performing the test on their own or those who may not have had a private place to test. Participants could return their test results in person at the distribution point, or via a WhatsApp message. A commitment to returning the result was not a prerequisite for study participation. Those who had a reactive test were offered confirmatory blood-based rapid detection HIV testing, in accordance with Zimbabwe National HIV testing guidelines, in a private tent at the distribution point. Those with a positive confirmatory test were referred for onward care to a clinic of their choice, with written referral letters. Linkage to care was defined as attending a clinic for antiretroviral therapy (ART) initiation for those who had a positive confirmatory blood-based HIV testing at a distribution point. This was confirmed through follow-up phone call to participants within 1 month of study participation.

### Data collection and statistical analysis

Sociodemographic data including age, sex, student residence on or off campus, access to income while studying, and cell phone ownership were obtained through an interviewer (research assistant) administered questionnaire, prior to the participant receiving the HIVST kit. HIV testing history was recorded. Audio computer-assisted self-interview recorded their sexual history, contraceptive use, circumcision history, and experience of gender-based violence.

Data were collected on electronic tablets using Open Data Kit and transferred to an MS Access database and analyzed using STATA, version 16.1 (STATA Corporation, College Station, Texas). Continuous variables were summarized as mean (standard deviation) or median (interquartile range [IQR]), and categorical variables as counts (percentages). The association between a priori defined variables (sex, age, prior knowledge of HIVST, living on campus, condom use at last sex and never having sex, having had an HIV test previously, having more than one sexual partner in the past year, being circumcised) and choosing to have HIVST off-site was determined using univariate logistic regression. Variables associated with the outcome at a p-value <.1 were taken forward into a multivariable model. This was analyzed for all participants for whom residential status was known (i.e., whether they lived on campus or not) (n = 5,138). Multiple imputation was used to impute missing data for residential status. In addition, we investigated the association among age, sex, college type, ever having sex, and previously having had a self-test and returning a test result for those who tested off-site using univariate logistic regression.

### Ethical considerations

Written informed consent was obtained from all participants. Ethical approval for the study was obtained from the Medical Research Council of Zimbabwe and the Biomedical Research and Training Institute Institutional Review Board. Permission to undertake the study was provided by the Ministry of Tertiary and Higher Education, Zimbabwe.

## Results

The distribution team spent 129 days in total across the 12 campuses with a median number of 7 (IQR 6–20) days on each campus. In total, 5,351 youth were given an HIVST with a median 32 (IQR 16–45) youth accessing HIVST per day. Patterns of testing uptake were similar across campuses with Tuesday and Thursday being the most popular day to access the service, and the initial days of the distribution campaign saw highest numbers of kits distributed, with numbers declining over time on campus (Figure A1).

### Participant characteristics

The median age of participants was 21 (IQR 20–23) years, 3,417 (64%) of whom were female. About a third of participants lived on campus and the vast majority–5,180 (97%)–owned cell phones, of which 4,893 (94%) were enabled for WhatsApp (Table 1).

More than 70% of participants reported having had sexual debut, with 1,799 (48%) having had two or more sexual partners in the last year. Of those who reported having had a sexual debut, 2,345 (62%) had used a condom at their last sexual intercourse (Table 1). Use of a female condom was uncommon. Hormonal contraception was currently used by 359 (17%) of sexually active females. Among those who reported sexual activity (n = 3,776), 129 (3%) said they had given gifts or money in exchange for sex, 100 (76%) of whom were male and 97 (3%) had received gifts or money in exchange for sex, 45 (46%) of whom were male. Although 1,577 (82%) males knew about VMMC as a method of HIV prevention, only 601 (31%) had been circumcised.

### HIV testing

Overall, 3,516 (66%) had previously tested for HIV a median of 2 (IQR 1–3) times. The majority had had their most recent test in a clinical facility, with only 16% having accessed HIV testing at a campus clinic. Less than half had heard of self-testing and 908 (17%) had previously used an HIV self-test kit, the majority having performed the test without provider assistance. For those who had never tested (n = 1,835), 154 (8%) had previously been offered an HIV test but declined.

**Figure 2:**
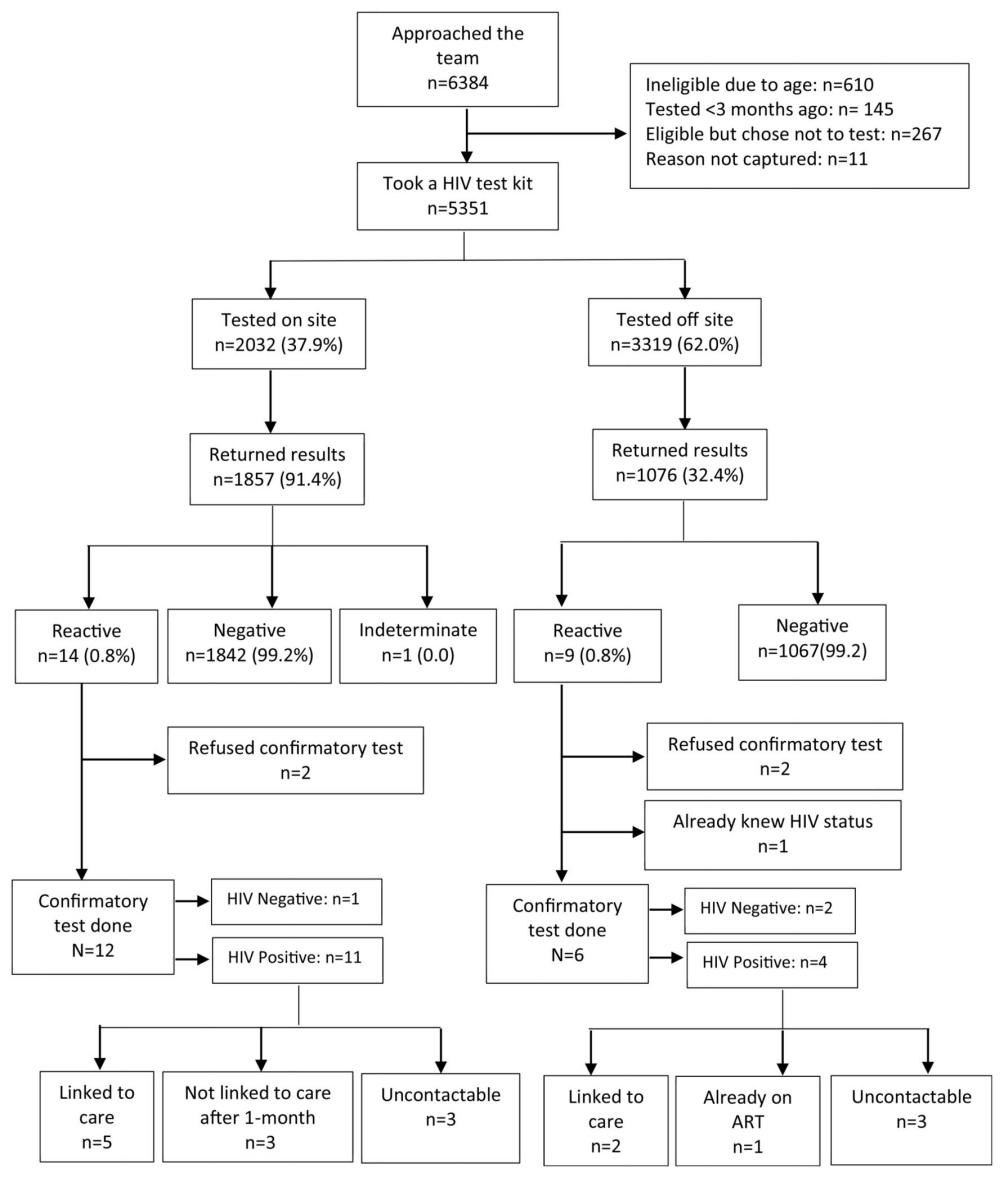
Flow diagram of recruitment, testing, and linkage to care.

Of the 5,351 participants, 3,319 (62%) opted to test off-site, the remaining tested on-site in a private booth (Figure 2). A slightly higher proportion of females–1,245 (36%)–than males–590 (30.5%)–were first time testers. On univariate analysis, living on campus (odds ratio [OR] 1.24, 95% confidence interval [CI] 1.10– 1.41, *p* < .01), having used a condom at last sexual intercourse (compared to having not used one) (OR 1.42, 95% CI 1.24–1.63, *p* < .001), never having had sex (OR 1.19, 95% CI 1.02–1.38, *p* = .024), having prior knowledge and awareness of HIVST (OR 1.23, 95% CI 1.10–1.38, *p* < .001), and being female (OR 1.12, 95% CI 0.99–1.26, *p* = .06) were associated with choosing to test off-site. On multivariate analysis, these factors remained independently associated with choosing to test off-site (Table 2). Sensitivity analysis using multiple imputation revealed similar results (not shown).

A total of 2,933 (55%) fed back their results, with 1,857 (91%) of those who tested on-site feeding back their results compared to 1,076 (32%) who tested off-site feeding back their results to the distribution team. In-person reporting was the preferred method of feedback, used by 2,683 (91%) of youth who fed back their result. Attending a vocational college and teachers training college compared to a university was associated with choosing to return results for those who tested off-site (OR 2.40, 95% CI 1.65–3.48, *p* < .001) (Table 3).

### HIV test results and linkage to care

Overall, 23 results were reported as reactive, 1 as indeterminate, and 2,909 as negative. Of those who tested off-site and returned results, nine had reactive results (1%) and for on-site testing, 14 had reactive results (1%) (Figure 2). Of the 23 participants with reactive results, 18 (78%) had confirmatory tests on-site, four youths refused confirmatory testing, and 1 knew his HIV positive status from a test done prior to study participation, and thus declined confirmatory testing. Fourteen of the confirmatory blood-based tests were positive, 3 (17%) were negative on both blood-based tests, and 1 was positive on the first bloodbased test and indeterminate on the second test. Repeat testing at a health facility confirmed a positive result.

Of the 15 participants whose confirmatory tests were positive, on follow-up phone call within 1 month, seven reported they had started ART, six of these seven having tested on-site. One reported that they had previously been aware their status was positive, having retested to confirm this, and was already on ART. Of the remaining seven, three said they had not started ART and four were unreachable by telephone.

## Discussion

This study investigated the feasibility of offering HIVST on campuses of higher and tertiary education institutions. It demonstrates that HIVST can reach a large number of youth, across a wide geographic distribution in a relatively short period of time using a small team of peer counsellors. Students who accessed the service represent the age group most at risk of acquiring HIV; in 2019, young people represented two out of every seven new HIV infections globally [13]. Although facility-based HIV testing was available at most of the colleges, one third of those who participated had never tested for HIV and of those who tested before few had used campus-based health facilities. Notably, we engaged youth at educational institutions to understand how to promote HIVST, and also engaged youth to deliver HIVST, both of which may have facilitated uptake [14].

Our qualitative exploration of this campus-based HIVST, reported elsewhere, showed that individuals found this approach was empowering as it promoted autonomy and was more convenient and socially acceptable among peers than facility- or other community-based HIV testing services [15]. Relatedly, another recent study that investigated HIVST in community-based testing showed that while HIVST offers autonomy, youth do value the support offered by providers and are fearful of losing this support if they test independently [16]. The flexible option of testing on-site or off-site offered in this study ensured that participants had access to post-test counselling and support, as well as autonomy and privacy if desired.

Less than half of participants had previously heard of HIVST but despite limited exposure to this new method, many opted to take the test home and not test on-site where assistance was available if needed. Those who had previously heard of HIVST had higher odds of testing off-site, likely reflecting more confidence in being able to perform the test independently as they had heard about it before. Those who lived on campus also were more likely to test off-site. It is possible that those who lived off-campus may have had less access to a private space to perform their HIVST. Those who had never had sex or used a condom at their last sexual encounter were also more likely to opt for off-site HIVST. A possible explanation is that they perceived themselves to be at lower risk of being HIV-positive and felt comfortable testing for HIV without support available. However, in the qualitative assessment of the process of offering HIVST on campus, some participants reported feeling “socially coerced” to test when other students were testing, while others felt there was insufficient privacy when testing on-site [15]. Factors such as these may have also contributed to participant choice of off-site testing, and further research investigating feasible, acceptable service delivery options for youth is needed.

Youth in sub-Saharan Africa remain at high risk of HIV infection. Despite the large number of HIV prevention and sexual and reproductive health programs in the region, the 2015 Zimbabwe Demographic and Health survey reported that only 55% of women and 56% of men aged 15–49 years [17] demonstrated comprehensive HIV knowledge. Notably, 40% of study participants reported that they had not used a condom at last sexual encounter, and only a third of males had been circumcised. Also, 35% of youth who accessed testing in this study had never tested for HIV highlighting the need for continued emphasis on accessible and acceptable HIV testing services for youth. Assessing possible exposure to HIV has previously been cited as a reason for accessing repeat HIVST among adults [14]. Increasing access to HIV testing, such as through the distribution of self-testing kits in locations convenient to the target group, may serve to “normalize” HIV testing and enable individuals to better monitor their HIV status in the context of ongoing risk.

HIV testing can serve as a gateway to accessing both HIV prevention and care services. This does require individuals to act upon their results and access appropriate services. In this, two issues arise: first, assessing linkage to care among those who choose to self-test can be challenging. The very ethos of HIVST is enabling individual autonomy, which conflicts with provider or community-led follow-up processes to ensure linkage to care which are inherently interpersonal endeavors. In our study, the majority of those who tested on-site fed back their results, but only a third of those who took away HIV kits to test off-site fed back their results. Therefore, it is difficult to assess both yield of testing and whether individuals accessed appropriate services in response to their test results. Second, while studies have shown a high uptake of HIVST, ensuring that individuals who self-test actually link to appropriate HIV prevention and care services remains a challenge [18–20]. In this study, just over half of those who had a confirmatory positive test had linked to HIV care at 1 month post diagnosis. The majority of those who had linked to care had tested on-site, and it is possible that the post-test counselling and support available to on-site testers may have facilitated linkage. We acknowledge that 1 month may be too short of a time period to adjust to a diagnosis of HIV and take action to initiate treatment, and thus our findings may not accurately reflect linkage to care for those with a positive test, which may have occurred at a later date. Measurement of linkage to care for HIV prevention services was not undertaken as this was outside of the scope of this study. It is critical that provision of HIVST is accompanied by provision of information on the importance of HIV prevention and care and guidance on how and where to access relevant appropriate prevention and care services.

Digital interventions may be an approach that could be combined with HIVST to facilitate linkage to care [14]. SMS reminders have been used to support adherence to treatment in HIV programs with some success [21–23]. Similar approaches could be used to increase awareness of HIVST, relay information about HIVST distribution drives in educational institutions and other sexual health interventions to increase awareness and access to health services. Cellphone ownership was very high in our target population and the majority of cellphones were enabled for WhatsApp, one of the most widely used social media platforms in Zimbabwe, and affordable. Digital interventions are particularly appropriate for youth and especially for those in higher education who are likely to have high levels of digital literacy.

Two of the 15 participants who tested HIV-positive had tested HIV-positive previously. They may have retested to confirm their HIV positive status or perhaps to check if they had been “cured” following HIV treatment. OMT tests may appear falsely negative in individuals taking ART [24]; this should be made explicit in addition to information about the need for confirmatory HIV testing with blood-based antibody tests for any reactive results.

The strengths of this study are the inclusion of different types and a wide geographic spread of tertiary level institutions. Both off-site and on-site testing was offered, which can inform future implementation models. We acknowledge several limitations. HIVST results were not available for nearly half of study participants and total positivity among testers was therefore not possible to ascertain. Few reactive results were reported and linkage to care was assessed at a single time point, making it difficult to ascertain linkage to care rates.

### Conclusion

In conclusion, provision of HIVST to youth on campus facilitates access to HIV testing and offers an opportunity to reach youth, who are at disproportionate risk of HIV infection but are a “hard-to-reach” group due perhaps to the lack of appropriate youth friendly peer-led activities to attract youth [25]. There is a need to increase awareness of HIVST and provision of self-testing kits should be coupled with guidance about HIV prevention and care services.

## Supplementary Material

Supplementary data related to this article can be found at https://doi.org/10.1016/j.jadohealth.2022.09.004.

Supplementary Figure 1

## Figures and Tables

**Table 1:** Participant characteristics (n = 5,351)

Variable	n(%)
Sociodemographic characteristics	
Sex, female	3,417 (63.8)
Age (years), median (IQR)	21 (20–23)
College/University	
University of Zimbabwe	1,308 (24.4)
Great Zimbabwe University	1,062 (19.9)
Masvingo Polytechnical College	262 (4.9)
Bindura University of Science & Education	758 (14.2)
Gweru Polytechnical College	670 (12.5)
Midlands State University	585 (10.9)
Belvedere Teachers College	72 (1.3)
Morgan Zintec Teachers College	65 (1.2)
Parirenyatwa School of Nursing	25 (0.5)
Kushinga Pikelele Polytechnical College	298 (5.6)
Young Africa Vocational Training College	102 (1.9)
Harare Institute of Technology	144 (2.7)
Residence on campus^a^	1,615 (31.4)
Currently in part-time employment	341 (6.4)
Income >300 USD per month	218 (4.1)
Living as married	339 (6.3)
Owns cellphone (either shared or personal phone)	5,180 (96.8)
Share ownership of phone with someone else	1,478 (28.5)
Sexual and reproductive health and substance use Ever had sex	3,776 (70.6)
If yes, no. of partners in the past month, median (IQR)	1 (0–1)
Used condoms at last sex (in sexually active)	2,345 (62.1)
Ever used female condom^b^	72 (3.3)
Have heard ofVMMC as a method to prevent HIV infection^c^	1,577 (81.6)
Have had voluntary medical male circumcision^c^	601 (31.1)
Currently use a hormonal family planning method^b^	359 (16.7)
Currently pregnant^d^	41 (1.2)
Ever received gifts of money for sex^e^	97 (2.6)
Ever given gifts of money for sex^e^	129 (3.4)
Been physically hurt or threatened to be physically hurt by current or previous partner^f^	80 (2.8)
Physically forced to have sex by current or previous partner^f^	120 (4.1)
Currently or ever used alcohol^f^	1,370 (47.2)
Currently or ever used drugs^f^	397 (13.7)
HIV testing history	
Previous HIV test	3,516 (65.7)
Location of most recent HIV test among previously HIV tested	
Clinic	1,509 (42.9)
Inpatient in hospital	295 (8.4)
During Pregnancy	35 (0.9)
Community based testing	454 (13.0)
Campus/College clinic	562 (16.0)
Private doctor	193 (5.5)
Other	468 (13.3)
Previously heard of HIV self-testing	2,358 (44.1)
Ever taken a HIV self-test	
Self-administered	678 (12.7)
Provider Assisted	230 (4.3)
Ever offered a test but declined^g^	154 (8.4)

IQR = interquartile range; VMMC = voluntary medical male circumcision.

a Data missing for n = 213.

b Female participants who said they were sexually active (n = 2,150).

c Male participants only.

d Female participants only.

e Denominator n = 3,776, those who stated they previously had sex.

f Denominator n = 2,904, as question was added later in the study.

g Denominator n = 1,835, i.e., those who have never had a test.

**Table 2:** Factors associated with choosing to test off-site (for those whose residential status is known), n = 5,138

	Total who tested off-site/total (%)	Univariate analysis odds ratio (95% confidence interval)	*p* value	Multivariate analysis adjusted odds ratio^a^ (95% confidence interval)	*p* value
Age (years)					
21–24	2,210/3,544 (62%)	0.96 (0.85–1.08)	.520	-	
16–20	1,009/1,594 (63%)	1.0			
Sex					
Female	2,078/3,267 (64%)	1.12 (0.99–1.26)	.06	1.16 (1.03–1.31)	.018
Male	1,141/1,871 (61%)	1.0	-	1.0	
Living on campus					
Yes	1,068/1,615 (66%)	1.24(1.10–1.41)	<.001	1.24 (1.09–1.40)	.001
No	2,151/3,523 (61%)	1.0		1.0	
Condom use at last sex					
Yes	1,504/2,277 (66%)	1.42 (1.24–1.63)	<.001	1.44 (1.26–1.65)	<.001
No	791/1,369 (58%)	1.0		1.0	
Never had sex	924/1,492 (62%)	1.19(1.02–1.38)	.024	1.16 (0.99–1.35)	.057
Previous HIV test					
Yes	2,101/3,349 (63%)	1.01 (0.90–1.14)	.864	-	
No	1,118/1,789 (63%)	1.0			
>1 Sexual partner in the past year					
Yes	1,323/2,111 (63%)	0.97 (0.85–1.12)	.688	-	
No	972/1,535 (63%)	1.0			
Never had sex	924/1,492 (62%)	0.94 (0.81 – 1.09)	.429	-	
Previously heard of HIV self-test					
Yes	1,476/2,257 (65%)	1.23 (1.10–1.38)	<.001	1.22 (1.09–1.37)	.001
No	1,743/2,881 (61%)	1.0		1.0	
Circumcision					
Yes	342/578 (59%)	0.89 (0.73–1.09)	.282	-	
No	799/1,293 (62%)	1.0			

a Factors associated with off-site testing on univariate analysis were included in multivariate analysis.

**Table 3:** Factors associated with choosing to return results for those who tested off-site (n = 3,319)

	Total who returned result/total tested off-site (%)	Univariate analysis Odds ratio (95% confidence interval)	*p* value
Age (years)			
21–24	715/2,277 (31.4)	0.86 (0.74–1.01)	.064
16–20	361/1,042 (34.6)	1.0	-
Sex			
Female	699/2,151 (32.5)	1.01 (0.87–1.18)	.898
Male	377/1,168 (32.2)	1.0	-
Type of educational institution			
University	712/2,255 (31.6)	1.0	
Polytechnical college	294/922 (31.9)	1.01 (0.86–1.20)	.863
Vocational and teacher’s college	62/118 (52.5)	2.40 (1.65–3.48)	<.001
School of Nursing	8/24 (33.3)	1.08 (0.46–2.54)	.854
Ever had sex		
Yes	151/428 (35.3)	0.93 (0.80–1.09)	.393
No	322/961 (33.5)	1.0	
Previous self-test			
Yes	754/2,358 (32.0)	1.03 (0.68–1.56)	.895
No	44/127 (34.6)	1.0	
