# Supplementary figures and images for: On Campus HIV Self-Testing Distribution at Tertiary Level Colleges in Zimbabwe Increases Access to HIV Testing for Youth
Source: J Adolesc Health. Author manuscript; Available in PMC 2023 Aug 11. (PMC7614942; doi:10.1016/j.jadohealth.2022.09.004)

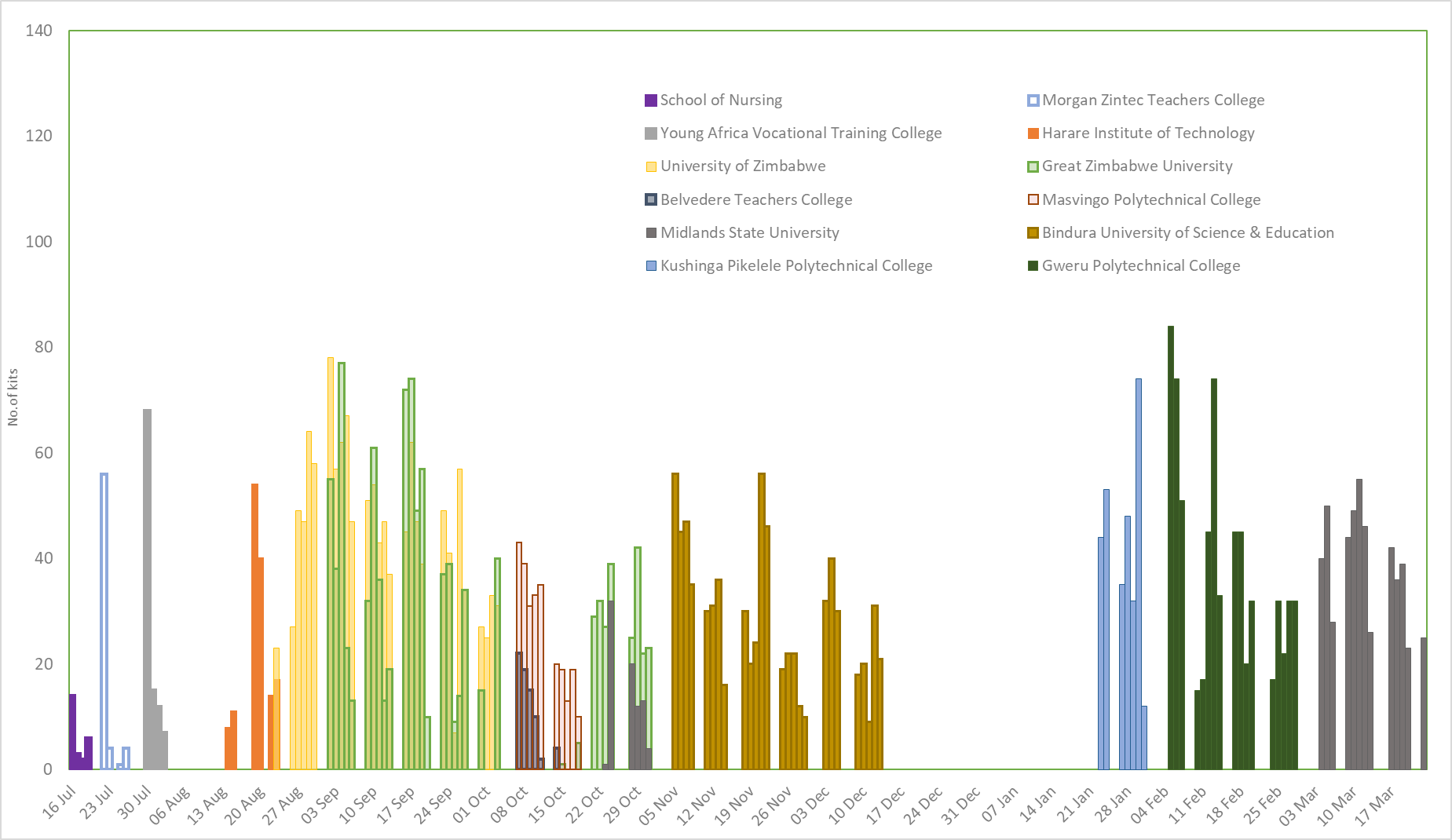

Supplement: Supplementary Figure 1 [file EMS182206-supplement-Supplementary_Figure_1.docx]
